# MEASURING ORAL HEALTH-RELATED QUALITY-OF-LIFE MEASURES IN ADULTS: DIRECTIONS FOR FUTURE APPLICATIONS

**DOI:** 10.1093/geroni/igae098.1430

**Published:** 2024-12-31

**Authors:** Zheng Zhu, Jing Wang, Bei Wu

**Affiliations:** New York University, New York, New York, United States; University of New Hampshire, Durham, New Hampshire, United States; New York University, New York, New York, United States

## Abstract

**Background:**

To identify the construct of oral health-related quality of life (OHRQoL) in published measures for adults and assess the psychometric properties of these measures.

**Methods:**

Nine databases were searched from inception of each database up to July 1st, 2023. Methodological quality was assessed by using the Consensus-based Standards for the Selection of Health Measurement Instruments (COSMIN) Risk of Bias Checklist. Thematic analysis was used to summarize all detected instruments, items domains and questions with regard to their wording, scales and the domains of OHRQoL. We used the COSMIN criteria to summarize and assess the psychometric properties of each PROM.

**Results:**

A total of 50 PROMs were identified. Six relevant domains that represent construct of OHRQoL: (1) pain/discomfort; (2) oral diseases and disorders; (3) functional limitation; (4) oral hygiene; (5) oral hygiene behavior; and (6) psychosocial impact. All studies were considered to have adequate methodological quality in terms of content validity, construct validity, and internal consistency. Limited information was retrieved on cross-cultural validity, criterion validity, reliability, hypothesis testing, and responsiveness. High-quality evidence on psychometric properties was provided for the oral health impact profile (OHIP), Geriatric Oral Health Assessment Index (GOHAI), and Oral Impacts on Daily Performances (OIDP).

**Discussion:**

We encourage the use of the identified six domains to enhance the development and adaptation of instruments for different health systems. OHIP, GOHAI, and OIDP are recommended for evaluating OHRQoL in adults, both in research and clinical practice, based on the specific aims of assessments and the response burden for participants.

